# Autophagy–Neuroinflammation Axis in Neurodegenerative Diseases: Mechanisms and Therapeutic Potential

**DOI:** 10.3390/cells15090813

**Published:** 2026-04-29

**Authors:** Liyuan Sun, Yong Zou, Lifeng Wang

**Affiliations:** 1 Hengyang Medical College, University of South China, Hengyang 421001, China; sly_july@163.com; 2 Academy of Military Medical Sciences, Beijing 100850, China

**Keywords:** autophagy, neuroinflammation, neurodegenerative diseases, mechanisms, therapeutic strategies

## Abstract

Neurodegenerative diseases, characterized by progressive neuronal loss and functional decline, impose a substantial global health burden. Autophagy, the principal intracellular degradative pathway for clearing misfolded proteins and damaged organelles, is vital for neuronal homeostasis, whereas maladaptive neuroinflammation is increasingly being recognized as a central driver of disease progression. A growing body of evidence indicates a bidirectional, tightly coupled relationship between autophagy and neuroinflammation: impaired autophagic flux promotes accumulation of damage-associated molecules that activate innate immune responses, while sustained inflammatory signaling further disrupts autophagy, together forming a self-reinforcing cycle that accelerates neurodegeneration. This interplay is regulated by diverse genetic, molecular, cellular, and environmental factors and manifests in cell-type-specific ways across microglia, astrocytes. Therapeutic strategies emerging from these insights include modulation of autophagic pathways (e.g., mTOR, AMPK, TFEB), targeted inhibition of inflammasome and pro-inflammatory mediators (notably NLRP3-related signaling), and delivery platforms for small molecules or nucleic acids, with increasing interest in multi-target and stage-specific interventions. This review integrates mechanistic evidence and translational advances, highlights gaps in cell-type and stage-specific understanding, and outlines priorities for developing safe, effective therapies that target the autophagy–neuroinflammation axis in neurodegenerative disorders.

## 1. Introduction

Neurodegenerative diseases are a diverse set of conditions defined by the progressive loss of neurons in the central or peripheral nervous system. The impairment of cognitive, memory, behavioral, sensory, or motor abilities, combined with the structural and functional breakdown of neural networks, significantly impacts the quality of life for tens of millions worldwide. These illnesses typically arise from a combination of genetic predispositions and anomalies in metabolic pathways. Core pathogenic features include cytoskeletal abnormalities, energy metabolism disorders, pathological protein aggregation, synapse and neural network dysfunction, and inflammatory responses, all of which ultimately contribute to neuronal death [1]. Among these, the accumulation of abnormal proteins not only damages neurons directly but also triggers chronic inflammatory responses by activating astrocytes and microglia [2]. The resulting inflammatory microenvironment further disrupts protein clearance processes, creating a vicious cycle [3,4]. In this context, autophagy, an essential intracellular degradation system, serves as a critical link between abnormal protein aggregation and neuroinflammation [5].

The term “autophagy” was coined by Belgian scientist Christian de Duve and colleagues following the discovery of lysosomes in 1956. Utilizing electron microscopy, they visualized autophagosomes, double-membrane structures within cells, that encapsulate cytoplasmic components [6]. Autophagy is a conserved metabolic process in eukaryotic cells wherein lysosomes degrade pathogens, abnormal protein aggregates, and damaged organelles. Based on the mechanisms of delivery to lysosomes, autophagy is categorized into macroautophagy, microautophagy, and chaperone-mediated autophagy [5]. Autophagy serves a dual function: it maintains cellular homeostasis and regulates cell death. It protects cells from oxidative stress and nutrient deficiency by generating energy through macromolecular breakdown, such as by eliminating damaged mitochondria to preserve genomic integrity. Conversely, excessive autophagy, for instance, as activated by the tumor suppressor gene DAPK, can induce type II programmed cell death under extreme stress [7]. In the nervous system, autophagy primarily functions to protect neurons through its precise control over intracellular protein synthesis and degradation. Specifically, it mediates the targeted destruction of misfolded proteins and damaged organelles, thereby preserving neuronal proteostasis by dynamically balancing synthesis and clearance mechanisms within neurons [8].

In the central nervous system, neuroinflammation is primarily mediated by glial cells, especially microglia and astrocytes. Neurons are considered important targets and secondary modulators of these processes. It is typically characterized by an imbalance between pro-inflammatory mediators, such as interleukin-1β (IL-1β) and tumor necrosis factor-α (TNF-α), and anti-inflammatory factors, such as TGF-β and IL-10. Persistent glial activation can promote neuronal injury and may also compromise blood–brain barrier integrity, thereby facilitating the entry of inflammatory mediators and immune cells into the central nervous system [9,10,11,12,13]. Under physiological conditions, neuroinflammation may support tissue repair and debris clearance; however, chronic or excessive activation of microglia and astrocytes can lead to sustained cytokine production, oxidative stress, and progressive neuronal damage. These inflammatory processes are strongly associated with neurodegenerative diseases, including Alzheimer’s disease and other related disorders [10,11,12,13].

Autophagy and neuroinflammation are not independent pathological processes but are interconnected through a bidirectionally regulated network. The autophagy–neuroinflammation axis appears to be a core pathogenic circuit in neurodegenerative diseases. Key elements of this crosstalk include the NLRP3 inflammasome, NF-κB signaling, and mitochondrial homeostasis. For example, activation of the NLRP3 inflammasome by abnormal proteins such as Aβ and α-synuclein, or by pathogenic stimuli such as LPS, can promote inflammatory cytokine production while suppressing autophagy-related proteins such as Beclin-1 and LC3-II [13]. Defects in autophagy then contribute to the accumulation of damaged mitochondria, which release mitochondrial DNA (mtDNA) and reactive oxygen species (ROS), further amplifying NLRP3 activation and inflammatory signaling [14]. Conversely, inflammatory signaling, often through NF-κB activation, can alter the expression of autophagy-related genes and impair cellular homeostasis. In glial cells, these reciprocal interactions may intensify inflammatory responses and worsen neuronal damage [15,16,17].

Consequently, interventions that modulate the autophagy–neuroinflammation axis, such as inhibition of the NLRP3 inflammasome or enhancement of autophagic flux, represent promising therapeutic strategies for neurodegenerative diseases. This review summarizes the reciprocal regulatory relationship between autophagy and neuroinflammation, outlines their roles in disease progression, and discusses recent therapeutic advances targeting this axis. We further highlight the need for multi-pathway intervention strategies and provide a framework for future research in neurodegenerative disorders.

## 2. Autophagy Dysregulation in Neurodegenerative Disorders

Cells detect perturbations in internal or external conditions, including oxidative damage, loss of growth factors, endoplasmic reticulum stress, or nutrient limitation, and respond by activating multiple intracellular signaling pathways. These signals initiate autophagy, a process tightly regulated by mTOR signaling. Under nutrient-rich conditions, mTORC1 inhibits the ULK1 complex, suppressing autophagy. In contrast, during starvation or stress, AMPK activates ULK1, triggering autophagosome formation [18]. Autophagosomes subsequently deliver cargo to lysosomes for degradation via LC3-mediated substrate recognition [19]. The ATG5-ATG12 conjugate and lipidated LC3-II are key protein markers indicative of autophagic activity [18]. Successful autophagic flux depends on lysosomal acidification and enzymatic function, highlighting the functional coordination between lysosomes and mitochondria.

Mitophagy, a selective form of autophagy that removes damaged or superfluous mitochondria, is increasingly being recognized as a central mechanism linking autophagy failure to neurodegeneration. The best-characterized pathway is the PINK1–PRKN/Parkin axis, which is activated by mitochondrial depolarization and promotes ubiquitin-dependent recruitment of autophagy adaptors. In addition, receptor-mediated mitophagy pathways involving BNIP3, BNIP3L/NIX, FUNDC1, OPTN, TBK1, and related molecules also contribute to mitochondrial quality control in neurons and glial cells. Defective mitophagy leads to accumulation of dysfunctional mitochondria, excess reactive oxygen species, mtDNA release, and downstream activation of inflammatory pathways. While general autophagy can be induced by mitochondrial release of ROS and mtDNA [20], deficits in autophagic flux, caused by lysosomal dysfunction such as LAMP2 deficiency or by mitochondrial injury, may promote accelerated neurodegeneration [19].

The brain is particularly reliant on the functional integrity of the autophagy–lysosomal pathway, a dependence that becomes more pronounced with aging [21]. Research indicates that age-related dysregulation of autophagy is closely linked to the pathogenesis of several neurodegenerative diseases, including Alzheimer’s disease, familial Parkinson’s disease, and amyotrophic lateral sclerosis (ALS). Deficits in autophagy, often stemming from mutations in autophagy-related genes that reduce lysosomal clearance capacity, play a major role in the pathogenic accumulation of aberrant protein aggregates and damaged organelles [22]. Furthermore, genetic studies have shown that mutations in various genes associated with neurodegeneration can specifically disrupt key stages of the autophagy process. These disruptions can occur at the level of signal initiation, substrate recognition and engulfment, autophagosome maturation and transport, or lysosomal degradation [21]. The efficient long-distance transport of autophagic cargo, which is critical for maintaining neuronal protein homeostasis, depends on a functional microtubule-based motor system and precise intracellular trafficking signals. A salient example is the retrograde transport of autophagosomes generated at distal synapses back to the lysosome-rich cell body for degradation. This intricate vesicular transport system requires properly coordinated energy metabolism and molecular labeling mechanisms to function correctly [23].

## 3. Neuroinflammation Triggers in Neurodegenerative Diseases

Neuroinflammation is widely recognized as a major contributor to the pathogenic progression of neurological disorders, particularly chronic neurodegenerative diseases. Beyond directly inducing neuronal degeneration, a persistent inflammatory milieu in the brain exacerbates disease-related clinical symptoms through complex molecular cascades. Although neurodegeneration arises from varied causes such as proteostatic imbalance and mitochondrial dysfunction, inflammation consistently represents a central and shared pathological feature across the disease trajectory. This reciprocal vicious cycle between inflammation and degeneration profoundly impacts glial activation states, synaptic plasticity, and blood–brain barrier permeability [24].

Neuroinflammation involves coordinated responses among immune cells, neurons, and inflammatory mediators, and it is largely driven by interactions among microglia, astrocytes, and the peripheral immune system [25]. Microglia, the resident innate immune cells of the central nervous system, are pivotal in initiating inflammatory responses. In response to injury or infection, they rapidly transition from a quiescent, ramified state to an activated phenotype [9]. This activation occurs when pattern recognition receptors (PRRs), such as Toll-like receptors (TLRs), recognize pathogen-associated or damage-associated molecular patterns (PAMPs/DAMPs) [10]. For instance, in Alzheimer’s disease, Aβ binds to microglial TLR4 and activates downstream NF-κB signaling, thereby promoting inflammatory cytokine production [9]. Similarly, traumatic brain injury induces the release of DAMPs such as HMGB1, which are recognized by TLR4 and RAGE and subsequently trigger NF-κB and MAPK pathways, leading to the release of TNF-α, IL-1β, and IL-6 [26]. Astrocytes are another essential component of neuroinflammation. Under pathological conditions, they become reactive, characterized by hypertrophy and increased glial fibrillary acidic protein (GFAP) expression, and release inflammatory mediators and neurotoxic compounds such as prostaglandins and nitric oxide [3,27]. The crosstalk between microglia and astrocytes is bidirectional and can amplify inflammatory signaling. In AD, for example, Aβ-activated microglia drive astrocytes toward a neurotoxic A1 phenotype, thereby exacerbating neuronal injury [10,27]. In addition, when the blood–brain barrier is compromised, peripheral immune cells such as T lymphocytes and macrophages can infiltrate the central nervous system and further intensify the inflammatory response [11].

Inflammatory mediators play a central role in neurodegenerative progression. Pro-inflammatory cytokines, particularly TNF-α and IL-1β, impair neuronal viability and function, with TNF-α inducing apoptosis and IL-1β disrupting neuronal development and synaptic formation [28]. These mediators also interfere with glutamate uptake, resulting in synaptic glutamate accumulation and excitotoxic neuronal damage [29]. Chronic inflammatory stimulation promotes neuronal loss through both apoptotic and necrotic mechanisms. In Alzheimer’s disease, this process contributes to the neuronal loss underlying memory impairment and cognitive decline [21]. Neuroinflammation also profoundly impacts synaptic integrity and myelin homeostasis. Inflammatory mediators can disrupt synaptic transmission, while immune-mediated damage to oligodendrocytes contributes to myelin breakdown and impaired neuronal signaling [22]. In parallel, activated microglia and astrocytes generate large amounts of reactive oxygen species (ROS) and nitric oxide (NO), causing oxidative damage and reinforcing inflammatory signaling [24]. The combination of ROS and NO generates peroxynitrite, which further exacerbates cellular injury. Elevated ROS and NO levels have been documented in the brains of AD patients, where they are associated with tau hyperphosphorylation and Aβ aggregation [24]. Chemokines also contribute to disease progression by recruiting peripheral immune cells to sites of pathology. In Parkinson’s disease, chemokines such as CCL2 attract T cells and monocytes to the brain, thereby amplifying neuroinflammation and neuronal degeneration [25]. Moreover, non-canonical pathways such as secretory autophagy (SA) have been implicated in inflammation-driven neurodegeneration. Excessive activation of SA, mediated through factors such as SKA2 and FKBP5, promotes NLRP3 inflammasome activation, Gasdermin D-mediated neurotoxicity, and IL-1β release, ultimately contributing to neuronal loss [26].

## 4. Autophagy–Neuroinflammation Interaction Mechanisms

**Figure 1:**
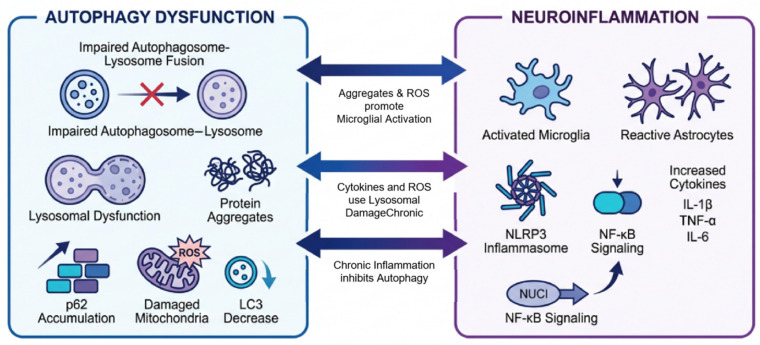
Schematic of the bidirectional regulation between autophagy dysfunction and neuroinflammation in neurodegenerative diseases. Arrows indicate the direction of regulation or signaling, double-headed arrows indicate bidirectional crosstalk, and the red cross mark denotes impaired or blocked fusion.

Growing evidence emphasizes that autophagy and neuroinflammation do not act in isolation but engage in complex bidirectional communication. This intricate reciprocal regulation underlies the conceptual and mechanistic framework of the autophagy–neuroinflammation axis. Dysregulation of this axis can give rise to a self-perpetuating vicious cycle that substantially drives the onset and progression of diverse neurological disorders. Consequently, dissecting the components and operational logic of this axis holds critical scientific and clinical value, as it is central to elucidating fundamental disease mechanisms and identifying novel therapeutic targets. In this section, we will examine in depth the core reciprocal regulatory mechanisms that constitute the axis and then analyze the key modulatory factors at the genetic, protein, cellular, and environmental levels that influence its function. As illustrated in Figure 1, autophagy and neuroinflammation are tightly interconnected and together form a self-amplifying pathogenic loop in neurodegenerative diseases.

### 4.1. Regulation of the Relationship Between Neuroinflammation and Autophagy

Autophagy regulates neuroinflammation through multiple mechanisms. Substantial evidence indicates that enhanced autophagic activity suppresses neuroinflammatory responses. In Alzheimer’s disease, for instance, autophagy activation has been shown to reduce the release of key inflammatory cytokines, including interleukin-1β (IL-1β) and tumor necrosis factor-α (TNF-α) [3]. Autophagy-mediated degradation of inflammasomes, which are multiprotein complexes critical for initiating and sustaining neuroinflammatory responses, represents a central mechanism. By recognizing and clearing these inflammasomes, autophagy inhibits the production and secretion of inflammatory cytokines. This anti-inflammatory role of autophagy is also supported by research on spinal cord injury, where autophagy induction attenuates neuroinflammation [27]. Consistently, defective autophagy exacerbates inflammatory responses, whereas its upregulation mitigates them. One specific pathway involves the clearance of damaged mitochondria via mitophagy, which reduces the release of mitochondrial damage-associated molecular patterns (DAMPs) that would otherwise trigger inflammation. The autophagy-related protein LC3, a central marker, undergoes lipidation from its cytosolic form (LC3-I) to a membrane-bound form (LC3-II), a process critical for autophagosome formation and maturation. Activation of the cannabinoid receptor type 2 (CB2R) significantly increases LC3 levels, indicating elevated autophagic flux. This upregulation of LC3 not only promotes autophagy but also facilitates the degradation of the NLRP3 inflammasome, thereby suppressing its activation. The critical role of autophagy in this process is further underscored by the fact that autophagy inhibitors (e.g., 3-MA) or lysosomal inhibitors (e.g., BafA1, CQ) block CB2R-mediated NLRP3 degradation [30]. Furthermore, autophagy modulates immune cell function by restraining excessive microglial activation and limiting the subsequent release of pro-inflammatory cytokines.

Conversely, neuroinflammation can exert significant influence over autophagic pathways. In an inflamed nervous system, cytokines such as TNF-α and IL-1β activate intracellular signaling cascades that modulate the initiation and progression of autophagy. Under certain conditions, this may represent a compensatory, protective response where induced autophagy helps mitigate inflammation-associated cellular damage by clearing dysfunctional organelles and proteins. However, in severe or sustained neuroinflammatory states typical of chronic neurodegenerative diseases, autophagy is frequently inhibited. Chronic inflammatory stimulation can lead to the aberrant expression or dysfunction of core autophagy-related proteins, thereby disrupting the entire autophagic process. Furthermore, neuroinflammation regulates autophagy by modulating its key upstream signaling hubs. Inflammatory milieus stimulate pathways like NF-κB and MAPK, which in turn can transcriptionally repress specific autophagy-related genes, effectively inhibiting autophagic activity [31].

Autophagy and neuroinflammation interact through a variety of signaling pathways, some of which critically regulate their interconnection. For instance, the Toll-like receptor (TLR) pathway functions as a key nexus in this regulatory network. TLR activation triggers downstream signaling that leads to NF-κB activation, which in turn promotes the synthesis and release of inflammatory cytokines, thereby driving neuroinflammation. Concurrently, TLR signaling can modulate autophagy by affecting the expression and activity of autophagy-related proteins. Another pivotal mechanism is the mammalian target of rapamycin (mTOR) signaling pathway, a major negative regulator of autophagy. Autophagy is initiated when mTOR activity is suppressed, whereas active mTOR inhibits autophagic processes. Beyond its direct control of autophagy, mTOR also indirectly influences microtubule dynamics by altering the phosphorylation state of tau and other microtubule-associated proteins [32]. Importantly, the production of inflammatory cytokines during neuroinflammation can stimulate the mTOR pathway, leading to autophagy suppression. Furthermore, the mTOR pathway indirectly fosters neuroinflammation by modulating immune cell metabolism and function [33].

### 4.2. Determinants of the Reciprocal Interplay Between Autophagy and Neuroinflammation

The interplay between autophagy and neuroinflammation is closely intertwined in the pathophysiology of neurological disorders, regulated by a multitude of factors. This section examines these modulating influences at the genetic, protein, cellular, and environmental levels.

#### 4.2.1. Genomic Factors

Genetic factors critically regulate the interplay between autophagy and neuroinflammation. Apolipoprotein E (APOE), encoded by the APOEgene, is integral to central nervous system function. The APOEε4 allele represents the strongest genetic risk factor for late-onset Alzheimer’s disease [34]. This variant disrupts multiple stages of autophagy, including autophagosome-lysosome fusion, and destabilizes lysosomal membranes in an allele-specific manner [35]. The resulting impairment in autophagic flux leads to intracellular accumulation of substrates, which in turn induces neuroinflammation. Furthermore, APOE ε4 may perturb neuronal cholesterol metabolism, creating a lipid environment that further inhibits autophagy and exacerbates inflammation. Brains of APOE ε4 carriers exhibit altered expression of autophagy-related proteins and elevated levels of pro-inflammatory mediators such as IL-1β and TNF-α [36]. Thus, APOE polymorphisms contribute to AD pathogenesis by concurrently dysregulating autophagy and promoting a neuroinflammatory state.

The TREM2 gene, expressed in myeloid cells including microglia, is another key regulator. Studies indicate that activation of the myeloid cell trigger receptor 2 (TREM2) enhances autophagic activity in microglia, which subsequently modulates inflammation-associated signaling pathways. Specifically, elevated autophagy downregulates the expression of Caspase Recruitment Domain-containing protein 9 (CARD9). This suppression of CARD9 attenuates downstream TLR4-mediated signaling, thereby reducing neuroinflammatory responses. Conversely, loss-of-function TREM2 mutations (e.g., R47H) or the absence of TREM2 impairs microglial autophagy. This impairment exacerbates neuroinflammation and hinders the clearance of β-amyloid. In summary, TREM2 activation promotes microglial autophagy, which in turn restrains TLR4-driven inflammation and improves cognitive outcomes in Alzheimer’s disease models. These findings elucidate a mechanism by which TREM2 modulates the autophagy-inflammation axis and provide a theoretical foundation for novel therapeutic strategies in AD [37].

Mutations in the TBK1gene, essential for both autophagy and innate immune responses, are associated with neurological disorders like amyotrophic lateral sclerosis (ALS). Studies using models with ALS-associated TBK1 loss-of-function mutations or conditional knockout in motor neurons demonstrate that reduced TBK1 kinase activity significantly impairs autophagy and modulates neuroinflammation [38]. TBK1 loss in motor neurons of SOD1 models accelerates disease progression, elevates SOD1 aggregation, and impairs autophagy, most likely because autophagy receptors are not phosphorylated and therefore cannot mediate effective substrate clearance. Interestingly, while TBK1 loss in motor neurons is detrimental, its absence in microglia may prolong survival in some models, potentially by attenuating interferon responses. This indicates that TBK1 dysfunction exerts complex, cell-type-specific effects on the autophagy–neuroinflammation axis during disease progression [38].

#### 4.2.2. Unbalanced Protein Homeostasis

Dysregulation at the protein level significantly influences the autophagy–neuroinflammation axis. Mutations in the superoxide dismutase 1 (SOD1) gene are closely linked to amyotrophic lateral sclerosis (ALS). Mutant SOD1 protein forms intracellular aggregates that not only are inherently toxic but also directly impair autophagy, primarily by blocking the fusion of autophagosomes with lysosomes [39]. This disruption leads to the accumulation of autophagic substrates, triggering intracellular stress and initiating neuroinflammation. ALS mouse models demonstrate increased inflammatory cytokine production and significant immune cell infiltration around mutant SOD1 aggregates, underscoring its central role in pathogenesis via concurrent disruption of autophagy and induction of neuroinflammation [40].

The expression of the key autophagy protein LC3 is directly regulated by the transcription factor FoxG1. Transcriptomic and validation studies show that FoxG1 binds to the LC3 promoter, repressing its transcription and thereby reducing both LC3 levels and autophagic activity. Notably, activation of the cannabinoid receptor type 2 (CB2R) decreases FoxG1 expression, weakens its binding to the LC3 promoter, increases LC3 synthesis, and consequently enhances the autophagic response [30].

Sirtuin 2 (SIRT2), a member of the NAD^+^-dependent deacetylase family, is another crucial regulator connecting autophagy, microtubule integrity, and inflammation. Studies establish a link between SIRT2 and FOXO1, a key modulator of autophagy. Under oxidative stress, the FOXO1-SIRT2 complex dissociates, enhancing FOXO1 acetylation and promoting autophagy induction. Concurrently, SIRT2 modulates the acetylation status of α-tubulin, affecting microtubule stability. SIRT2 mutant mice exhibit mitochondrial abnormalities, elevated oxidative stress, reduced ATP production, and failure of the autophagy–lysosome pathway, alongside increased acetylation of mitochondrial proteins. Thus, in Alzheimer’s disease contexts, SIRT2 indirectly regulates autophagy by modulating both FOXO1 activity and α-tubulin acetylation, thereby linking microtubule dynamics to autophagic function [41].

Amyloid proteins play a well-established role in neurodegenerative diseases. Research using a mouse model of mucopolysaccharidosis type IIIA (MPS-IIIA) revealed progressive intraneuronal accumulation of various amyloid species, including tau, prion protein (PrP), Aβ, and α-synuclein, alongside neurodegeneration. These proteins exhibit pathogenic synergy. For instance, α-synuclein promotes the fibrillation of the two primary AD-related proteins, tau and Aβ. Studies using double-transgenic models confirm that Aβ accelerates α-synuclein fibrillation both in vitro and in vivo, while α-synuclein can induce abnormal aggregation of Aβ1-38. Furthermore, tau and α-synuclein demonstrate a cross-nucleation effect, each initiating and accelerating the other’s fibrillation in a synergistic cascade that drives aberrant protein aggregation [42,43,44,45]. The aggregation of these amyloids impairs lysosomal function, which in turn slows autophagic flux and delays the clearance of toxic materials. The resulting lysosomal dysfunction and protein accumulation trigger neuroinflammatory reactions. This link is evident in AD brains, where extensive microglial activation and inflammatory cytokine release are observed surrounding Aβ plaques, indicating that amyloid-driven disruptions in autophagy contribute significantly to neuroinflammation and disease pathogenesis [46].

#### 4.2.3. Interactions Between Neurons and Microglia

Neurohomeostasis in the nervous system depends on tightly regulated interactions between neurons and microglia. In neurodegenerative diseases, neuronal damage triggers the activation of microglia, which in turn impacts neuronal health through the release of inflammatory mediators. Research utilizing mouse models of Huntington’s disease (HD) has revealed a specific communication pathway mediated by chemokine receptor 5 (CCR5), which is expressed on both cell types. In HD mice, activation of surface CCR5 on microglia initiates signaling to neurons that induces autophagic activity. However, as the disease progresses, this autophagy induction becomes progressively dysregulated. The resulting excessive autophagy ultimately contributes to neuronal damage. Concurrently, activated microglia release substantial amounts of inflammatory cytokines, including TNF-α and IL-1β, exacerbating neuronal injury by fostering a neuroinflammatory milieu. This interplay implies that neuron–microglia crosstalk orchestrates both autophagy and neuroinflammation and that its dysfunction may actively promote the pathogenesis of neurodegenerative diseases [47].

#### 4.2.4. Metabolic and Environmental Factors

Metabolic and environmental factors also play a major role in the pathophysiology of neurodegenerative diseases by modulating the autophagy–neuroinflammation axis. Reactive oxygen species (ROS) inhibit autophagy by degrading key proteins like Beclin-1 while concurrently activating the NLRP3 inflammasome. Infections and pathogen-associated molecular patterns (PAMPs), such as lipopolysaccharide (LPS), suppress autophagy by triggering microglial inflammation via TLR4 signaling. Age-related declines, particularly in lysosomal function, impair autophagic clearance and promote the accumulation of inflammatory mediators. Furthermore, senescent cells exhibiting the senescence-associated secretory phenotype (SASP) release factors like IL-6 and IL-8, which both inhibit autophagic activity and activate glial cells [48].

**Figure 2:**
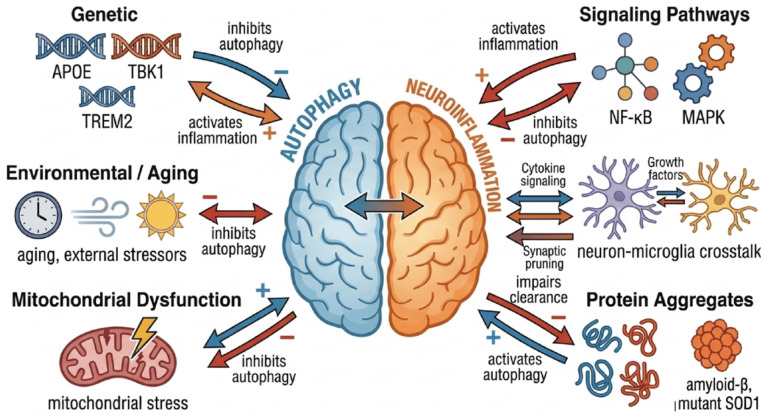
Factors Influencing the Interaction of Autophagy and Neuroinflammation. Autophagy and neuroinflammation are centered as two interconnected pathogenic processes. Blue elements and arrows denote autophagy-related processes, whereas orange elements and arrows denote neuroinflammation-related processes. The color of each directional arrow corresponds to the process being promoted or inhibited. Solid arrows indicate regulatory direction or signaling flow; double-headed arrows indicate reciprocal crosstalk between autophagy and neuroinflammation; + denotes activation or promotion; − denotes inhibition or suppression.

In summary, the interplay between autophagy and neuroinflammation is regulated by a complex network of genetic, environmental, molecular, and cellular factors. Genetic variations in loci such as APOE, TBK1, and TREM2 critically influence both autophagic and inflammatory responses. In addition, environmental stressors and aging can impair autophagy, while mitochondrial dysfunction and pathological protein aggregates further exacerbate autophagic failure and neuroinflammatory activation. At the cellular level, neuron-microglia crosstalk and inflammatory signaling pathways, including NF-κB and MAPK, form a tightly interconnected regulatory axis that links autophagy dysfunction to neuroinflammation. Together, these processes create a self-amplifying vicious cycle that contributes to neurodegenerative progression (Figure 2).

## 5. Contributions of Autophagy and Neuroinflammation to Neurodegenerative Disorders

The interplay between autophagy and neuroinflammation exerts a critical but context-dependent influence across neurodegenerative diseases, with its relative importance and functional consequences varying by disease. This complex relationship has long been a research focus and is increasingly recognized for its therapeutic relevance. In this section, we analyze the distinct contributions and reciprocal regulation of autophagy and neuroinflammation in the pathogenesis of several major neurodegenerative disorders.

### 5.1. Alzheimer’s Disease (AD)

Alzheimer’s disease (AD) is a progressive neurological disorder characterized by cognitive decline and is the most prevalent form of dementia. Epidemiological estimates indicate that approximately 50 million individuals worldwide are affected by it, and this number is expected to rise to 152 million by 2050 as the population ages [49]. The substantial disability burden and irreversible brain damage associated with AD impose considerable socioeconomic costs. In the United States, the cost of AD and related dementia care is projected to reach 360 billion in 2024 and exceed 2.8 trillion globally by 2030 [50]. In China, AD accounts for 60–80% of dementia cases and is a leading cause of disability and mortality among the elderly, affecting approximately 3% of adults aged 65 and older [51]. Therefore, elucidating the pathophysiology of AD remains critical for understanding disease progression and identifying pathogenic mechanisms.

The defining pathological hallmarks of AD are the extracellular accumulation of β-amyloid (Aβ), derived from amyloid precursor protein (APP) [52,53,54], and the intracellular aggregation of hyperphosphorylated microtubule-associated protein tau. Insufficient clearance of Aβ and hyperphosphorylated tau leads to the formation of amyloid plaques and neurofibrillary tangles, respectively [55]. Activated microglia cluster around Aβ plaques and release pro-inflammatory mediators such as IL-1β, which further promotes tau phosphorylation and aggravates neuronal injury. Beyond protein aggregation, AD pathogenesis also involves altered calcium homeostasis, synaptic loss, mitochondrial dysfunction, and sustained microglia-mediated inflammation, all of which contribute to progressive neurodegeneration.

A prominent feature of AD is impairment of autophagy, which has been observed in patient brain tissue and is increasingly recognized as a contributor to disease progression rather than merely a secondary consequence. Electron microscopy studies have revealed abnormal accumulations of lysosomes, autophagosomes, and vacuolar structures at synaptic terminals, as well as autophagic vesicles containing cathepsin within neuronal cytoplasm [56]. These findings indicate a failure in the late stages of autophagic clearance. Consistently, the downregulation of multiple autophagy-related proteins in AD brains suggests that autophagy dysfunction is actively involved in disease progression. Several AD risk genes, including PSEN1, PICALM, CLU, and TREM2, are known to regulate autophagic flux [57], further supporting the close relationship between defective proteostasis and AD pathology. In particular, impaired autophagic activity can aggravate the accumulation of both Aβ and tau, thereby intensifying the vicious cycle between defective protein degradation and neuronal injury.

Autophagy also plays a critical role in regulating neuroinflammation. In AD brains, the buildup of hyperphosphorylated tau and Aβ is tightly linked to autophagic dysfunction. Astrocytes participate in Aβ clearance through autophagy, a process regulated by LC3B and SQSTM1. Inhibition of autophagy increases mitochondrial oxidative stress, which in turn promotes amyloid plaque formation [58]. Key autophagy proteins, including Beclin1, VPS34, and ATG5, are essential for recycling Aβ receptors and supporting plaque elimination. Moreover, studies in animal models have shown that knockout of autophagy-related genes such as Rubicon and ATG5 increases tau hyperphosphorylation, indicating that autophagy is also important for preserving tau homeostasis [59].

Under physiological conditions, tau binds to and stabilizes microtubules; however, in AD, hyperphosphorylated tau detaches from microtubules and forms insoluble fibrillar deposits. These pathological aggregates impair axonal transport and disrupt neuronal architecture. Autophagy can counteract this process by targeting aggregated tau for degradation, whereas autophagy inhibition further enhances tau accumulation and neuronal damage [60]. Experimental evidence supports this relationship, as hyperphosphorylated tau has been shown to colocalize with LC3-positive autophagosomes [61]. In addition, increased autophagosomes in the hippocampus of AD patients are accompanied by reduced lysosomal enzyme activity, indicating a blockade in the terminal stage of autophagic flux [19]. In Alzheimer’s disease, mitophagy is frequently impaired in both neurons and glial cells, contributing to mitochondrial dysfunction, oxidative stress, and inflammatory activation. Reduced PINK1/Parkin-mediated clearance of damaged mitochondria can promote Aβ- and tau-associated toxicity, while defective receptor-mediated mitophagy involving BNIP3, NIX, or FUNDC1 may further compromise mitochondrial homeostasis. These defects are particularly relevant in microglia and astrocytes, where impaired mitophagy may exacerbate inflammatory signaling and reduce the capacity for debris clearance [62,63].

Neuroinflammation is now recognized as the third major pathological hallmark of AD, following Aβ deposition and neurofibrillary tangles [9]. Activated microglia and astrocytes, together with elevated levels of pro-inflammatory mediators such as TNF-α, IL-1β, and reactive oxygen species (ROS), are commonly found surrounding amyloid plaques and neurofibrillary tangles composed of hyperphosphorylated tau [64]. Although neuroinflammation initially contributes to the clearance of accumulated Aβ, it also generates cytotoxic compounds that paradoxically promote further Aβ deposition and ultimately drive neurodegeneration [65]. In neurons, TNF-α activates the JNK pathway, which upregulates both BACE1 and GSK-3β, thereby exacerbating tau phosphorylation and increasing Aβ production [3]. In addition, ROS can oxidatively modify Aβ, enhancing its aggregation propensity. These inflammatory and oxidative events collectively worsen synaptic dysfunction and neuronal loss.

Astrocyte reactivity is another important contributor to AD pathology. AD induces significant transformations in astrocytes and promotes their conversion into reactive A1-type astrocytes. These A1 astrocytes secrete inflammatory mediators and neurotoxic compounds, such as CHI3L1 and complement proteins, which impair neuronal development and survival [11,27]. Elevated markers of A1 astrocytes have been detected in the brains of AD patients, highlighting their contribution to disease progression. Beyond microglia and astrocytes, peripheral immune cells also participate in AD-related neuroinflammation. T cells and B cells can cross a compromised blood–brain barrier, where T cells secrete cytokines that alter microglial and astrocytic activation states, while B cells produce antibodies against antigens such as Aβ, forming immune complexes that further amplify neuroinflammation [10]. Thus, central and peripheral immune responses jointly shape the inflammatory environment in AD.

Autophagy and neuroinflammation are two closely interconnected processes in AD pathogenesis [66]. Activated microglia accumulate around amyloid plaques and release inflammatory mediators such as TNF-α and IL-1β. IL-1β suppresses autophagy through mTOR activation, while inflammation-induced defects in lysosomal acidification can paradoxically promote autophagosome accumulation [9]. The resulting impairment in autophagic flux diminishes the clearance of both tau and Aβ, thereby worsening pathogenic protein deposition. Furthermore, Aβ stimulation triggers the assembly of the NLRP3 inflammasome, which promotes the maturation and release of IL-1β. This process not only strengthens neuroinflammation around plaques but also interferes with autophagy-mediated tau clearance. In addition, glia maturation factor (GMF)-regulated NLRP3 activation is thought to influence the efficiency of abnormal protein removal by affecting autophagosome–lysosome interactions. Conversely, autophagic abnormalities hinder degradation of the NLRP3 inflammasome, thereby amplifying inflammatory signaling and establishing a vicious cycle; indeed, inhibition of NLRP3 in animal models reduces plaque burden by approximately 40% [67].

Several layers of disease heterogeneity should also be considered when interpreting preclinical and clinical findings. Substantial differences exist between animal models and human AD in both the immunological microenvironment and pathogenic progression. For example, the phagocytic efficiency of Aβ is significantly impaired by the TREM2 R47H mutation, and this effect is further complicated by species-specific differences in microglial gene expression profiles between rodents and humans. Such discrepancies may limit the translatability of findings related to the autophagy–inflammation axis and bias efficacy predictions for therapies targeting this interaction, including anti-NLRP3 approaches [68,69]. In addition, AD pathogenesis is dynamic and exhibits stage-specific inflammatory signatures and autophagic activity during early Aβ deposition and late tau propagation. Mechanistically, tau can exacerbate neuroinflammation through a GSK-3β-mediated positive feedback loop, whereas Aβ oligomers can activate TLR4/NF-κB signaling to upregulate BACE1 expression and further increase Aβ production. The TREM2 R47H mutation also impairs lysosomal acidification in microglia and reduces autophagic flux efficiency. These observations underscore the importance of gene–environment interactions in AD initiation and progression [69].

### 5.2. Parkinson’s Disease (PD)

Parkinson’s disease (PD) is a progressive neurodegenerative disorder characterized by the aggregation of -synuclein and the loss of dopaminergic neurons in the substantia nigra. Its clinical manifestations include motor symptoms such as bradykinesia, resting tremor, and rigidity, as well as non-motor symptoms including depression and cognitive decline [70,71]. A core pathogenic feature of is the abnormal aggregation of -synuclein into Lewy bodies, which can activate the NLRP3 inflammasome in microglia. This activation promotes the release of IL-1β and exacerbates neuronal damage [72]. In addition to protein aggregation, pathogenesis involves mitochondrial dysfunction, oxidative stress, lysosomal impairment, and persistent neuroinflammation, all of which contribute to progressive dopaminergic neurodegeneration.

Autophagy deficiencies are intimately linked to α-synuclein pathology in PD. Chaperone-mediated autophagy (CMA) is a primary pathway for α-synuclein degradation; decreased expression of its key receptor, LAMP2A, leads to the accumulation of toxic oligomers. Research indicates that the ADCY-PKA signaling cascade is downregulated via the neuronal LAMP2A-lysosomal pathway, which induces complex acute neurodegenerative and neuroinflammatory responses following metabolic stress [73]. Furthermore, studies demonstrate that LAMP2A prevents the accumulation of the autophagy marker p62 under acute oxidative stress, suggesting it enhances autophagic flux and promotes neuroprotection [74]. Extracellular α-synuclein can itself inhibit autophagy by activating microglial TLR4, suppressing the critical autophagosome protein LC3-II, and upregulating p62. Conditional deletion of the essential autophagy gene Atg5in microglia exacerbates neuroinflammation and neuronal death in α-synuclein overexpression models, confirming that α-synuclein suppresses autophagy via the TLR4-p38/Akt-mTOR pathway [75]. Additionally, mutations in the GBA gene reduce glucocerebrosidase activity, impairing the autophagy–lysosomal pathway (ALP) and compromising α-synuclein clearance [76]. Autophagic deficits are further aggravated by LRRK2 mutations, which alter lysosomal function through aberrant phosphorylation of Rab proteins [77,78].

Mitophagy, the selective autophagic removal of mitochondria, is also disrupted in PD through the PINK1/Parkin pathway. This dysfunction results in the accumulation of reactive oxygen species (ROS), which can activate the NLRP3 inflammasome, intensifying inflammation and promoting dopaminergic neuronal death [20,77]. Autophagy dysfunction thus creates a deleterious cycle by allowing toxic metabolites to accumulate and preventing the clearance of damaged organelles. Conversely, activated microglial autophagy may counteract inflammation by degrading the NLRP3 inflammasome and phagocytosing extracellular α-synuclein [79]. However, inflammatory cytokines such as IL-1β can in turn suppress autophagy via the mTOR pathway, thereby reinforcing a positive feedback loop that sustains disease pathology. Genetic vulnerabilities, such as SNCA multiplication, together with environmental toxins, such as MPTP, can disrupt autophagic homeostasis and facilitate the pathological spread of α-synuclein. In MPTP-induced PD models, elevated markers of autophagy, including LC3B and Beclin1, have been observed, indicating an activated but likely dysregulated autophagic response. The concomitant increase in LC3B together with the loss of tyrosine hydroxylase-positive neurons suggests that excessive or inefficient autophagy may also contribute to neuronal damage, highlighting the context-dependent role of autophagy in PD pathogenesis [80,81,82].

Autophagy is strongly linked to microglial activation in PD and plays an essential role in controlling neuroinflammation. Its dysfunction in microglia can contribute to PD-like pathology. Knockdown of the core autophagy gene Atg5 specifically in mouse microglia results in the loss of tyrosine hydroxylase (TH)-positive neurons, increased neuroinflammation, reduced striatal dopamine levels, and impaired motor coordination and cognitive learning. This autophagic deficiency facilitates activation of the NLRP3 inflammasome through the PDE10A-cAMP signaling pathway. Atg5 deletion markedly elevates PDE10A protein levels without altering its mRNA expression, indicating post-translational regulation. Inhibition of autophagy lowers intracellular cyclic adenosine monophosphate (cAMP) levels, which in turn promotes NLRP3 inflammasome activation [83]. Thus, autophagy defects in microglia not only impair protein and organelle clearance but also amplify inflammatory signaling cascades that worsen dopaminergic neuronal loss.

Neuroinflammation constitutes another critical factor in the pathophysiology of PD. α-Synuclein oligomers activate the M1 phenotype of microglia via TLR2/4, leading to the release of pro-inflammatory molecules such as TNF-α and IL-1β. In vitro studies demonstrate that α-synuclein preformed fibrils (PFFs) activate the NLRP3 inflammasome in microglia, a process dependent on ASC speck formation and caspase-1 cleavage [79]. Conversely, functional autophagy can inhibit NLRP3 activity by removing damaged mitochondria. The inhibition of autophagy by α-synuclein promotes NLRP3 assembly by increasing mitochondrial ROS production and mtDNA release [77]. In astrocytes, loss of ATP13A2 function leads to NLRP3 overactivation, driving astrocyte-mediated neuroinflammation [84]. Mutations in ATP13A2 (PARK9) impair polyamine transport, resulting in intracellular polyamine accumulation. This exacerbates neuroinflammation by promoting mitochondrial stress and α-synuclein aggregation, while metabolites such as acrolein directly damage lysosomal membranes and further strengthen the self-perpetuating inflammatory cycle [85].

Emerging research also implicates the gut–brain axis in PD pathogenesis. Dysbiosis may facilitate the transmission of α-synuclein aggregates from the gut to the central nervous system, while TLR signaling in the intestine amplifies local and systemic inflammation [71,86]. In parallel, defective mitophagy, particularly through inactivation of the PINK1/Parkin pathway, leads to mitochondrial injury and mtDNA release. This can activate the cGAS-STING pathway, initiating a type I interferon response and promoting the release of IL-6 and TNF-α. Damaged mitochondria also generate additional ROS, creating a self-perpetuating cycle linking autophagy impairment, inflammation, and oxidative stress [87,88].

### 5.3. Huntington’s Disease (HD)

Huntington’s disease (HD) is an autosomal dominant neurodegenerative disorder caused by a CAG repeat expansion in the HTT gene. It is clinically characterized by motor abnormalities, cognitive decline, and psychiatric symptoms. The pathogenic core of the disease involves the aggregation of aberrant mutant huntingtin protein (mHTT), which drives both neuroinflammation and neuronal death. In HD, autophagy and neuroinflammation interact closely and critically influence disease progression [87].

A vicious cycle is established in which mHTT accumulation directly impairs autophagy. This impairment manifests as disrupted Rab protein function, inhibited autophagosome–lysosome fusion, and the consequent accumulation of toxic proteins [1]. Conversely, functional autophagy promotes the degradation of mHTT and helps maintain intracellular proteostasis. Defective autophagic clearance therefore contributes to progressive neuronal injury and exacerbates the accumulation of pathogenic aggregates. In addition to its effect on protein turnover, mutant huntingtin can impair mitochondrial homeostasis and interfere with mitophagy, thereby contributing to energy failure and neuronal degeneration. Dysregulation of mitophagy-associated signaling may also affect glial support functions and aggravate inflammatory responses in the diseased brain [88].

Two primary drivers in the pathophysiology of HD are glial hyperactivation and dysregulation of the NLRP3 inflammasome pathway. The aggregation of mHTT induces mitochondrial dysfunction, leading to the generation of reactive oxygen species (ROS) and potassium (K+) efflux. These events activate the NLRP3 inflammasome, which enhances neuroinflammation and promotes the maturation of pro-inflammatory cytokines such as IL-1β and IL-18 [89]. In parallel, oxidative stress triggers the translocation of high-mobility group box 1 (HMGB1) from the nucleus to the cytoplasm. Cytosolic HMGB1 can bind to mHTT, facilitate its nuclear import, and simultaneously amplify inflammatory signaling through receptors such as TLR4 and RAGE. HMGB1 also inhibits autophagy, thereby establishing a detrimental inflammation–autophagy imbalance axis [26]. Furthermore, microglia-derived chemokines CCL-3, CCL-4, and CCL-5 can bind to and activate neuronal CCR5 receptors. This activation facilitates mTORC1 signaling, which in turn interferes with autophagic flux and aggregate clearance. A self-sustaining loop may be formed as CCR5-mediated autophagy inhibition impedes the degradation of CCR5 itself, contributing to its persistent overexpression [29].

Environmental factors, such as exposure to heavy metals, may also indirectly modulate the autophagy–neuroinflammation network by influencing glial cells and the gut microbiota. Studies have shown that heavy metals such as iron and manganese can exacerbate HD pathogenesis by inducing inflammatory glial phenotypes and gut dysbiosis. These factors activate the microglial NLRP3 inflammasome through oxidative stress and mitochondrial damage, while also suppressing the expression of autophagy-related proteins. In HD patients, gut dysbiosis, including an altered Firmicutes/Bacteroides ratio, is associated with reduced levels of beneficial short-chain fatty acids (SCFAs), increased blood–brain barrier permeability, and aggravated neuroinflammation [90]. Such changes suggest that peripheral metabolic and microbial disturbances may further intensify central pathological processes in HD.

Overall, the pathogenesis of Huntington’s disease involves a complex and interconnected network of autophagy impairment and neuroinflammation. The convergence of mutant protein aggregation, mitochondrial dysfunction, inflammasome activation, glial reactivity, and gut–brain axis disturbance collectively drives disease progression. However, the spatiotemporal dynamics of key molecules in this interplay, such as NLRP3 and HMGB1, throughout HD progression remain poorly understood, highlighting the need for further investigation into the molecular mechanisms underlying HD pathology.

### 5.4. Other Neurodegenerative Diseases

Beyond the three representative disorders discussed above, a range of other neurodegenerative diseases also exhibit substantial overlap in pathogenic mechanisms, particularly with respect to protein aggregation, autophagy impairment, mitochondrial dysfunction, oxidative stress, glial activation, and chronic neuroinflammation. In amyotrophic lateral sclerosis (ALS), degeneration of both upper and lower motor neurons is closely associated with defective autophagy–lysosomal pathways, including dysregulation of TBK1, optineurin, TDP-43, and C9orf72, which collectively impair aggregate clearance and promote neuronal loss [91]. Neuroinflammation is also prominent in ALS, with microglial and astrocytic activation and elevated pro-inflammatory mediators such as TNF-α, IL-18, NOD2, and Spp1 [92].

Similarly, multiple-system atrophy (MSA), frontotemporal dementia (FTD), progressive supranuclear palsy (PSP), corticobasal degeneration (CBD), and Lewy body dementia (LBD) all show varying degrees of dysfunction in proteostasis and inflammatory regulation. MSA is characterized by widespread α-synuclein deposition, glial cytoplasmic inclusions, proteasome impairment, defective autophagic flux, mitochondrial abnormalities, and NLRP3-mediated neuroinflammation. FTD involves mutations in C9orf72, GRN, TRIM21, TREM2, and SQSTM1, which disrupt autophagy, lysosomal function, and glial homeostasis, thereby facilitating TDP-43 and tau pathology together with inflammatory amplification [93,94]. PSP and CBD, as major tauopathies, are characterized by tau aggregation, mitochondrial dysfunction, oxidative stress, and glial activation, with evidence that impaired autophagy contributes to abnormal tau accumulation and neuroinflammatory signaling [95,96,97,98,99,100]. CBD additionally involves defective proteasomal and lysosomal pathways, cGAS-STING activation, and complement-mediated synaptic pruning [101]. LBD, which includes both dementia with Lewy bodies and Parkinson’s disease dementia, is marked by α-synuclein aggregation, reduced expression of autophagy-related genes such as BECN1 and LC3B, lysosomal dysfunction, mTOR hyperactivation, and NLRP3-driven inflammation [102]. Although these diseases differ in their dominant pathogenic proteins, neuronal vulnerability, and clinical presentation, they converge on a common mechanistic framework in which defective autophagy and persistent neuroinflammation mutually reinforce each other and accelerate neurodegeneration.

## 6. Therapeutic Developments Targeting the Autophagy–Neuroinflammation Axis

In neurodegenerative diseases, autophagy dysfunction and neuroinflammation establish a self-perpetuating “autophagy–inflammation” vicious cycle. Impaired autophagic flux leads to the accumulation of misfolded proteins and dysfunctional mitochondria, which release damage-associated molecular patterns (DAMPs) such as Aβ and α-synuclein. These DAMPs activate the NLRP3 inflammasome in microglia, driving an overactive inflammatory response. This inflammatory milieu, in turn, further impedes autophagic clearance through mechanisms such as the cleavage of Beclin-1 and the inhibition of transcription factor EB (TFEB) nuclear translocation, thereby accelerating neuronal death. Consequently, disrupting this detrimental interplay represents a principal therapeutic objective. This section will focus on the latest advances in treatment strategies that target the connection between autophagy and neuroinflammation [103].

### 6.1. Pharmacological Modulation of the Autophagy–Neuroinflammation Axis

Modulating autophagic activity represents a promising therapeutic strategy for limiting neuroinflammation, as autophagy is a key regulator of inflammatory homeostasis. A number of pharmacological agents that regulate autophagy have demonstrated anti-inflammatory and neuroprotective properties [104].

In Parkinson’s disease, rapamycin and its derivatives, such as everolimus, enhance autophagy primarily by inhibition mTORC1, thereby reducing α-synuclein accumulation [75,77]. mTORC1 Inhibition has also been associated with increased Beclin-1 expression and reduced levels of inflammatory cytokines such as IL-1β and TNF-α; however, excessive autophagosome accumulation suggests that late-stage autophagic flux may remain incomplete under some conditions [105]. In ALS, clinical studies have shown that rapamycin may modulate regulatory T cells and suppress IL-18 signaling [106].

Although primary therapeutic endpoints were not fully achieved, some patients exhibited improvements in inflammatory markers. In addition, antibody-based therapies targeting repeat-associated non-AUG RAN proteins derived from the expanded C9orf72 repeat can reduce aggregation of autophagy-associated proteins and alleviate neurodegeneration in animal models [107]. These findings suggest that simultaneously modulating both inflammatory and autophagic processes represents a viable treatment strategy for ALS.

Several agents directly targeting neuroinflammatory pathways also exert effects on autophagic regulation. For example, the NLRP3 inhibitor MCC950 and anti-synuclein antibodies such as prasinezumab have shown potential in attenuating neuroinflammation and reducing pathogenic protein burden [75,77]. Beyond classical autophagy modulators, GLP-1 receptor agonists such as exenatide have demonstrated neuroprotective effects in clinical studies. In parallel, gene-based restoration of lysosomal function, including AAV- mediated GBA delivery, has advanced to early clinical testing. Additional strategies aimed at lowering neurotoxicity include inhibition of polyamine metabolism, such as DFMO, or targeting the polyamine transporter ATP13A2 [76].

Natural compounds and repurposed agents have also attracted considerable attention. Polyamines such as spermidine can induce autophagy and exert anti-inflammatory and neuroprotective effects [108]. Spermidine has been reported to reduce neuroinflammation and improve cognitive performance in animal models of Alzheimer’s disease. Trehalose, an mTOR-independent autophagy inducer, enhances autophagic flux, suppresses inflammatory cytokine production, and promotes functional recovery in experimental models of spinal cord injury [39,40]. The “molecular tweezer” CLR01 acts as a broad-spectrum inhibitor of amyloid self-assembly. In mouse models of mucopolysaccharidosis type IIIA (MPS-IIIA), CLR01 treatment successfully decreased lysosomal expansion, restored autophagic flux, and inhibited amyloid formation. This effect is attributed to CLR01’s ability to bind lysine residues, thereby disrupting aberrant intermolecular connections during amyloid aggregation and reducing amyloid-induced interference with lysosomal and autophagic activity. The restored autophagy effectively cleared toxic aggregates and intracellular waste, reduced neuroinflammation, and improved memory deficits. CLR01-treated animals exhibited decreased inflammatory cytokine expression, reduced cerebral amyloid deposition, and normalized expression/activity of autophagy-related proteins, indicating that CLR01 alleviates neuropathology by regulating autophagy [57]. The natural compound crocin markedly upregulates the expression of several autophagy-related proteins, including LC3B-II, ATG5, and ATG7. LC3B, a crucial marker and component of autophagosomes, participates in their assembly and transport. Crocin promotes autophagy primarily by activating the AMPK pathway via STK11/LKB1. AMPK activation not only enhances the transcription of autophagy-linked genes but also helps maintain cytoskeletal integrity by modulating microtubule dynamics. For instance, AMPK inhibits the mTORC1 complex, which negatively regulates autophagy and influences microtubule stability. Therefore, AMPK pathway activation supports microtubule network integrity, facilitating efficient autophagosome trafficking and enhancing the degradation of toxic substances like Aβ [109]. Kai-Xin-San (KXS), a traditional formulation, has been found to enhance LC3B expression and promote its colocalization with microtubule-associated proteins. KXS reduces neuroinflammation by inhibiting NLRP3 inflammasome activation and promoting mitochondrial clearance via the PINK1/Parkin-mediated mitophagy pathway. This discovery expands our understanding of the link between microtubules and mitophagy and suggests novel therapeutic avenues for AD [110]. Similarly, berberine modulates neuronal autophagy by altering the levels of autophagy-associated proteins, specifically the microtubule-associated protein LC3B and others like ATG16L and ATG7. The IGF-regulated JNK-AKT signaling pathway may be instrumental in this effect [111].

Inhibiting neuroinflammation can potentially be a therapeutic strategy to enhance autophagy because it reduces autophagy function. Anti-inflammatory medications can reduce inflammatory reactions in neurological conditions, which will return autophagy to normal. Nonsteroidal anti-inflammatory medicines (NSAIDs), for instance, can decrease neuroinflammation, limit cytokine production, and hence improve autophagy function in neurodegenerative diseases [112].

### 6.2. Microbiota-Based and Gut–Brain Axis Therapies

Accumulating evidence suggests that the human gut microbiota contributes directly and indirectly to the pathogenesis of central nervous system disorders through modulation of the gut–brain axis, GBA. Under physiological conditions, the gut microbiota helps maintain intestinal immune homeostasis, epithelial barrier integrity, and metabolic balance. However, excessive proliferation of harmful bacteria and the accumulation of their byproducts can lead to gut dysbiosis, which disrupts the structural integrity of both the intestinal mucosal barrier and the blood–brain barrier, BBB. This barrier dysfunction facilitates the translocation of microbial components and immune mediators, thereby promoting abnormal immune cell infiltration or accumulation within the central nervous system. As a consequence, regulatory T cell function is suppressed, microglial homeostasis is disturbed, and a persistent neuroinflammatory state is established. This dysbiosis-driven pathological process may further accelerate the aggregation of misfolded proteins, while chronic inflammation in turn enhances the spread of these pathogenic proteins to neighboring neurons, thereby creating a self-perpetuating vicious cycle that contributes to neurodegeneration [113,114].

Among gut microbiota-derived metabolites, short-chain fatty acids SCFAs, including butyrate, propionate, and acetate, are particularly important in Alzheimer’s disease, AD, because of their ability to regulate both autophagy and neuroinflammation. These metabolites are produced by bacterial fermentation of dietary fiber and help preserve intestinal barrier function while also influencing the central nervous system through the GBA. Butyrate, one of the most well-studied SCFAs, exerts anti-inflammatory effects by inhibiting histone deacetylases HDACs, upregulating anti-inflammatory mediators such as IL-10, and suppressing the NF-κB pathway, thereby reducing the production of pro-inflammatory cytokines including IL-1β and TNF-α [115]. In addition, SCFAs activate G protein-coupled receptors GPR41/43, which helps maintain immune homeostasis in both intestinal and central immune cells, including microglia, thereby limiting excessive inflammatory activation. Simultaneously, SCFAs can enhance autophagic activity through the AMPK/mTOR pathway, promoting the clearance of abnormal protein aggregates such as Aβ and α-synuclein. They also facilitate a shift in microglia toward an anti-inflammatory phenotype and attenuate inflammatory responses in reactive astrocytes. Experimental studies have further supported these effects; for example, butyrate supplementation has been shown to attenuate neuroinflammation, protect dopaminergic neurons, and inhibit TLR4/NF-κB signaling, thereby improving motor deficits in Parkinson’s disease models [116].

Importantly, SCFAs may also indirectly regulate brain inflammation and autophagy through gut hormones such as GLP-1 and vagal nerve signaling. Clinical studies have shown that patients with AD exhibit reduced SCFA levels, which are associated with decreased gut microbial diversity, whereas probiotic interventions can restore SCFA levels and ameliorate disease-related pathology. Therefore, strategies aimed at restoring gut microbial homeostasis, including dietary fiber supplementation, probiotic therapy, prebiotics, and other microbiota-targeted interventions, may represent promising therapeutic approaches for neurodegenerative diseases by modulating the autophagy–inflammation network [117].

### 6.3. Combined Therapeutic Strategies

Given the reciprocal relationship between autophagy dysfunction and neuroinflammation, combination therapy is likely to be more effective than monotherapy. Concurrent modulation of both processes may produce synergistic benefits by simultaneously reducing protein aggregation, limiting inflammatory signaling, and restoring cellular clearance mechanisms. For instance, co-activation of autophagy and the antioxidant NFE2L2/NRF2 pathway provides better relief in neuropathic pain models than activation of either pathway alone, as these pathways jointly reduce oxidative stress and inflammation [118]. In Alzheimer’s disease, combining an autophagy activator with an anti-inflammatory agent may more effectively reduce both Aβ deposition and neuroinflammation, leading to greater cognitive improvement than either treatment separately [44]. This combination approach represents a promising frontier, though further research is needed to optimize specific regimens and thoroughly evaluate their long-term safety and efficacy.

### 6.4. Novel Intervention Paradigms

Recent advances in therapeutic development have expanded beyond conventional inhibitory strategies toward interventions that directly eliminate pathogenic proteins, damaged organelles, or upstream disease drivers. Among these, targeted degradation technologies and gene therapy represent two promising paradigms for neurodegenerative disease treatment. However, successful translation of these approaches depends not only on target specificity and biological efficacy, but also on the development of efficient delivery systems capable of overcoming the blood–brain barrier, BBB, and achieving sufficient exposure in relevant central nervous system, CNS, regions [119].

#### 6.4.1. Autophagy-Targeting Degradation Systems

Autophagy-targeting chimeras, AUTACs, represent a novel degradation strategy that hijacks the autophagic machinery. An AUTAC molecule typically consists of a target-binding ligand linked to a degradation tag that recruits autophagosome membranes, thereby facilitating selective autophagic clearance of the target. In neurodegenerative diseases, AUTACs could be designed to target pathogenic proteins such as mutant SOD1 or TDP-43, potentially offering greater specificity than conventional inhibitory approaches. Unlike proteolysis-targeting chimeras PROTACs, which recruit E3 ubiquitin ligases and promote proteasomal degradation, AUTACs can also remove larger structures such as damaged mitochondria and protein aggregates through autophagy [120]. Similarly, autophagy-tethering compounds, ATTECs, are bifunctional molecules that simultaneously bind a target protein, such as mutant huntingtin, and an autophagy-related protein, such as LC3. By physically linking pathogenic substrates to the autophagic machinery, ATTECs promote selective degradation independent of the ubiquitination pathway. These compounds have shown efficacy in preclinical models of Huntington’s disease and may also be applicable to the clearance of damaged mitochondria, suggesting potential value in Parkinson’s disease and other disorders characterized by proteostatic and organelle dysfunction [121,122].

Despite their conceptual appeal, the clinical translation of AUTACs and ATTECs remains challenging. Their relatively large molecular size, chemical complexity, and limited brain penetrance may restrict delivery efficiency, while poor pharmacokinetic properties and off-target degradation could compromise safety. In addition, sustained activity in the brain may require repeated administration or specialized carriers, further complicating clinical development. To address these limitations, engineered exosomes, lipid nanoparticles, and polymer-based nanocarriers are being explored as delivery vehicles for autophagy-modulating cargo, including rapamycin and autophagy-regulating miRNAs such as miR-124, directly to neuronal tissue. In parallel, autophagy peptide-conjugated gold nanoparticles have been developed to enhance Aβ clearance in Alzheimer’s disease models, highlighting the potential of nanotechnology-assisted autophagy modulation [123]. Nevertheless, the efficiency, biocompatibility, biodistribution, and large-scale manufacturability of these systems remain major barriers to clinical application.

#### 6.4.2. Gene Therapy

Gene therapy provides another innovative intervention paradigm by enabling sustained modulation of disease-related genes rather than transient symptomatic relief. In neurodegenerative diseases, this strategy can be used to silence pathogenic genes, restore deficient protective factors, or deliver therapeutic proteins to affected neurons. Viral vectors, particularly adeno-associated viruses AAVs, are widely used because of their favorable neuronal tropism and relatively durable expression profile [124].

For example, gene-silencing approaches based on RNAi or antisense oligonucleotides can reduce the production of toxic proteins such as mutant huntingtin, mutant SOD1, or α-synuclein. Conversely, gene replacement or gene augmentation strategies may restore neuroprotective genes involved in proteostasis, mitochondrial maintenance, synaptic function, or autophagy regulation. In addition, gene-editing technologies such as CRISPR/Cas systems offer the possibility of directly correcting pathogenic variants at the DNA level, although safety, off-target effects, and delivery efficiency remain major concerns [125,126].

A major obstacle for gene therapy is the efficient and cell-specific delivery of genetic material across the BBB. Although AAV vectors, engineered exosomes, and receptor-mediated transport systems have shown promise, each platform has important limitations, including restricted cargo capacity, immunogenicity, heterogeneous distribution within the brain, and difficulty achieving controlled long-term expression. Moreover, repeated vector administration may be limited by pre-existing or treatment-induced immune responses. These issues are particularly important in chronic neurodegenerative disorders, in which durable and safe gene delivery is required over extended periods. Therefore, continued progress in vector engineering, cell-type-specific promoters, and non-viral delivery platforms will be essential for improving the feasibility of gene therapy as a disease-modifying strategy [127].

#### 6.4.3. Delivery Considerations for Clinical Translation

In addition to platform-specific limitations, several general delivery challenges should be considered when evaluating the translational potential of autophagy-based therapies. First, the BBB remains the principal barrier for both small molecules and biologics, often resulting in insufficient drug concentrations at the disease site. Second, even when brain entry is achieved, regional heterogeneity in disease pathology may limit the effectiveness of systemically administered agents. Third, many delivery systems that perform well in animal models show reduced efficiency in humans because of species differences in vascular architecture, immune surveillance, and brain size. Finally, safety concerns such as immunogenicity, unintended tissue accumulation, toxicity, and poor biodegradability may restrict long-term use [128,129].

Accordingly, future delivery strategies will likely require a combination of approaches, including receptor-mediated transcytosis, focused ultrasound-assisted delivery, exosome-based transport, and biomaterial-guided release systems. The development of disease-relevant delivery platforms that achieve robust brain penetration, cell-type selectivity, and favorable safety profiles will be a prerequisite for translating these emerging therapies into clinical practice [130].

### 6.5. Limitations and Future Directions

Although substantial progress has been made in targeting the autophagy–neuroinflammation axis, several important challenges remain. First, many pharmacological candidates have shown promising effects in preclinical models, but their efficacy and safety still require confirmation in larger and more rigorous clinical trials. Second, the precise molecular mechanisms by which genetic background, disease stage, and cell-type specificity shape therapeutic responsiveness remain incompletely understood. Third, because autophagy is a fundamental homeostatic process, excessive or poorly timed activation may have unintended consequences, including incomplete flux or cellular stress.

A major challenge in translating these findings from animal studies to human clinical applications is the substantial difference between experimental models and human physiology. Most current neurodegenerative disease models, particularly transgenic mouse models, reproduce only selected pathological features, such as protein aggregation, gliosis, or motor impairment, but fail to fully capture the complex and heterogeneous course of human disease. Species differences in brain anatomy, immune system function, lifespan, metabolism, and blood–brain barrier integrity further limit the predictive value of preclinical results. In addition, treatment responses observed in animal models may not directly reflect human pharmacokinetics, safety profiles, or clinically meaningful outcomes. Therefore, although animal studies provide essential mechanistic insights and proof-of-concept evidence, their therapeutic relevance should be interpreted with caution, and future studies should prioritize more translationally relevant models, including human induced pluripotent stem cell-based systems, organoids, and large animal models [131].

Future studies should therefore prioritize the development of disease-stage-specific interventions, biomarker-guided patient stratification, and combination strategies that integrate autophagy modulation, anti-inflammatory therapy, and upstream approaches such as microbiota regulation or targeted degradation. A better mechanistic understanding of the autophagy–neuroinflammation crosstalk will be essential for translating these emerging therapies into clinically effective treatments.

## 7. Prospects

The reciprocal regulation between autophagy and neuroinflammation holds significant therapeutic potential for neurodegenerative diseases. However, its complex mechanisms and context-dependent effects necessitate further elucidation. A deeper understanding of this interaction is crucial for developing effective treatments. Although recent research has begun to unravel the molecular links between autophagy and neuroinflammation, many signaling pathways and regulatory factors remain unexplored. Employing cutting-edge techniques, such as gene editing, proteomics, and other multi-omics approaches, will be essential to identify key regulatory nodes and to map the comprehensive signaling networks that govern this interplay. Furthermore, the development of novel drug delivery systems promises to accelerate the translation of these therapeutic insights into clinical applications. An important and unresolved question concerns the stage-specific roles of neuroinflammation and autophagy. Their precise functions and relative contributions likely differ between the early initiation and late progression phases of neurodegenerative diseases. Therefore, comparative studies analyzing the distinct features and mechanisms of the autophagy–neuroinflammation axis across various disorders will form a critical foundation for precision medicine. By resolving these spatiotemporal dynamics and mechanistic details, future research can pave the way for targeted, combinatorial therapies that effectively disrupt the pathogenic cycle between impaired autophagy and chronic neuroinflammation.

## Data Availability

No new data were created or analyzed in this study.
